# A Study on Mesoporous Silica Loaded With Novel Photosensitizers HCE6 and Oxaliplatin for the Treatment of Cholangiocarcinoma

**DOI:** 10.3389/fonc.2021.665182

**Published:** 2021-06-29

**Authors:** Pei-Jian Zhang, Meng-Dong Liu, Fang-Yong Fan, Ke-Xia Liu

**Affiliations:** ^1^ Department of General Surgery Endoscopy, Cangzhou Central Hospital, Cangzhou, China; ^2^ Department of Pain, Cangzhou People’s Hospital, Cangzhou, China; ^3^ Department of General Surgery, Huanghua People’s Hospital, Huanghua, China

**Keywords:** HCE6, oxaliplatin, mesoporous silica nanoparticles, cholangiocarcinoma, photodynamic therapy

## Abstract

**Purpose:**

Cholangiocarcinoma (CCA) is a malignant tumor with a high incidence. The therapeutic effect of conventional chemotherapy and radiotherapy is not obvious. Photodynamic therapy (PDT) is an ideal modality to fight cancer, and the nature of photosensitizer limits its application in clinical therapy. The aim of this study was to explore a novel mode of drug delivery for the intervention of bile duct cancer.

**Methods:**

Oxaliplatin and photosensitizer HCE6 were loaded with mesoporous silica nanoparticles (MSNs) to synthesize Oxaliplatin/HCE6-MSNs (OH-MSNs); the structure of OH-MSNs was characterized by transmission electron microscopy (TEM) and dynamic light scattering (DLS), the drug release rate was detected by high performance liquid chromatography; the cellular activity, apoptosis level, and the expression levels of intracellular apoptosis and autophagy-related factors of OH-MSNs on cholangiocarcinoma cells were observed by CCK-8, flow cytometry, colony formation assay, and Western blot; the effects of OH-MSNs on cholangioma growth were observed by mouse tumor formation, immunohistochemistry, and tissue Tunel staining.

**Results:**

The release of OH-MSNs to Oxaliplatin was enhanced under acidic conditions; compared with Oxaliplatin or O-MSNs, OH-MSNs showed more potent killing effects against cholangiocarcinoma cells (P<0.05), and exerted notably inhibitory effects on the activity of cholangiocarcinoma cells (P<0.05), promoted their apoptosis (P<0.05), and greatly facilitated the expression of pro-apoptotic factors and autophagic factors in cholangiocarcinoma cells (P<0.05), and markedly inhibited the expression of anti-apoptotic factors and autophagic inhibitory factors (P<0.05); moreover, OH-MSNs could significantly suppress the growth of mouse cholangiocarcinoma (P<0.05) and induce apoptosis of tumor cells compared with Oxaliplatin or O-MSNs (P<0.05).

**Conclusion:**

MSNs loading greatly increases the killing effect of Oxaliplatin on cholangiocarcinoma cells and upgrades the autophagic level of cholangiocarcinoma cells, while OH-MSNs synthesized by further loading HCE6 have a more apparent killing effect on cholangiocarcinoma cells.

## Introduction

Cholangiocarcinoma (CCA) is a malignant tumor that arises from bile duct epithelium. Thanks to its special location, the early symptoms of CCA are not obvious, and the tumor has usually developed to the middle and advanced stages when patients experience evident adverse reactions, resulting in missing the optimum treatment period (1, 2). Oxaliplatin serves as one of medicines commonly used in clinical practice for the treatment of bile duct cancer. However, CCA is insensitive to conventional chemotherapy and radiotherapy. Patients are mostly characterized by poor prognosis, short survival, and high mortality through clinical intervention, and the 5-year survival rate of patients who underwent radical surgery for bile duct carcinoma remains unsatisfactory (3, 4). Thus there is an urgent need for an effective therapeutic strategy for clinical intervention in bile duct cancer. For the past few years, with the constant improvement of the overall medical level worldwide, minimally invasive therapies such as photodynamic therapy (PDT) have received considerable attention in the treatment of bile duct cancer.

PDT is an emerging anti-cancer method in which photosensitizers that accumulate at the tumor site under the excitation of specific wavelengths of light react with oxygen and water to form free radicals, thereby killing cancer cells by means of oxidative stress (5, 6). It is favored by investigators because of its relatively few side effects and higher targeted treatment in relative to conventional chemoradiotherapy. Nevertheless, the fact that photosensitizers are highly hydrophobic makes their transport in patient more difficult, triggering certain limitations in PDT clinically (7, 8). To address this situation, to improve the efficiency of drug delivery can has a positive impact on the anti-cancer effect of photosensitizers. Currently, drug delivery methods such as micelles and liposomes have been successively used to improve the delivery efficiency of photosensitizers (9, 10).

Autophagy is a vital physiological process in cells and an essential mechanism to maintain cell survival (11). Generally, cells maintain their homeostasis by a certain level of autophagic response to refrain themselves from toxic substances-induced damage, while excessive autophagy may also give rise to apoptosis (12, 13). In most tumors, tumor tissues exhibit significant autophagic activity that is regulated by a variety of tumor-associated signaling pathways. Autophagy both inhibits and promotes tumors during tumorigenesis and progression, which has been generally recognized (14). Increased autophagic response may both increase and attenuate resistance to chemotherapeutic agents, depending on the characteristics of the tumor itself and the duration of the stress (15). Autophagic cell death is thought to result from the induction of chemotherapeutic agents in mammalian cells, and the induction of tumor cell death by triggering excessive or sustained autophagy has also been deemed as a potential strategy for tumor therapy (16, 17).

In this study, we proposed to use mesoporous silica nanoparticles (MSNs) loaded with both novel photosensitizer HCE6 and first-line therapy medicine (Oxaliplatin) for cholangiocarcinoma to combine chemotherapy with PDT and observe its intervention effect on cholangiocarcinoma.

## Material and Methods

### Synthesis of OH-MSNs

To obtain MSN, 0.3 mL of ammonia solution (25 wt.%) and 1 mL of 0.15M cetyltrimethylamine bromide (CTAB) solution were added to the water while stirring. After 5-10 min, 0.5-2.0 mL of APTES solution (10 μL APTES dissolved in 2 m L ethanol) and 0.5-2.0 m L of tetraethyl orthosilicate (TEOS) solution (60 μL TEOS dissolved in 0.5 m L ethanol) were alternately added to water with continuous stirring for 5 h. Finally, the CTAB in the solution was removed by reflux at 60°C with a solution of ethanol (60 mL) and ammonium nitrate (0.3g) for three times. Afterwards, the Oxaliplatin (0.5 mmol) and HCE6 (0.5 mmol) were mixed with MSN (0.1 mmol) in 15 ml deionized water. The mixture was then sonicated for 30 minutes, and centrifuged to exclude the free Oxaliplatin and HCE6.

### Structural Characterization of OH-MSNs

The morphology of OH-MSNs was scanned using a Tecnai G2 20 S-TWIN transmission electron microscope (TEM, Philips, Netherlands). OH-MSNs were stained with 1% uranyl acetate solution and examined for their micellar morphology by a 200 K vacuum accelerated voltage. The zeta potential of OH-MSNs was detected by dynamic light scattering (DLS) at 25°C using a Zetasizer Nano ZS (Malvern, UK).

### Assay of Drug Release Kinetics

Assay of loading capacity and encapsulation efficiency: the supernatant of OH-MSNs was collected, shaken well, and the content of Oxaliplatin in OH-MSNs was measured by high performance liquid chromatography. Chromatographic column: Shimadzu C18 (250mm * 4.6mm,5 μm); mobile phase: methanol:water (10:90, V/V); flow rate: 1.0 mL/min; detection wavelength: 250 nm; injection volume: 20 μL, column temperature: 25°C. The content of HCE6 in Oxaliplatin/HCE6-MSNs was determined by fluorescence spectrophotometer. The absorbance of Oxaliplatin/HCE6-MSNs was measured at a wavelength of 652 nm with Oxaliplatin/MSNs as a control. The loading capacity (LC) and encapsulation efficiency (EE) of OH-MSNs were calculated as follows:

EE%=Wt/Ws×100%

LC%=Wt/Wo×100%

Notes: Wt: mass of Oxaliplatin encapsulated in the nanoparticles; Wo: initial dose of Oxaliplatin; Ws: total mass of the nanoparticles after lyophilization.

Assay of drug release profile: The *in vitro* drug release was investigated by the dialysis bag method. The OH-MSNs nanoparticles solution was dispersed with 1 mL of PBS containing 0.1% Tween 80 (pH 4, 5, 6.5 or 7.4) in a dialysis bag, placed in a centrifuge tube containing 30 mL of the corresponding release medium, and subjected to *in vitro* release assay at 100 rpm on a 37°C shaker. 200 μL of release medium was collected at 0.5, 1, 2, 3, 4,6, 8, 12, 24, 48 and 72 h and replenished with an equal volume of fresh release medium. As above, the concentration of released drug was detected by fluorescence and HPLC methods, respectively, and the cumulative release was calculated.

### Cell Culture

FRH0201 and HuCCT1 cells in bile duct cancer (Shanghai Tongpai Biotechnology Co., Ltd., China) were used as the study subjects. Cells were cultured using high-sugar DMEM (Thermo Fisher Scientific, USA) with 10% calf serum. All cells were cultured in a constant temperature incubator at 37°C and 5% CO_2_, and cells were passaged when they reached 90% polymerization.

### Cell Activity Assay

FRH0201 and HuCCT1 cholangiocarcinoma cells in good condition were selected and grown in 96-well plates, and the corresponding drugs were added to wells at a concentration of 10 ug/ml Oxaliplatin and 1 ug/ml HCE6, respectively. 12h later, cells were irradiated with 652 nm laser at 2-8 mW/cm^2^ for 5 min, and then incubated in an incubator for 24 h. Finally, cell viability was measured using CCK8. CCK-8 assay: 10 μL of CCK-8 solution was added to the cells to be tested in a 96-well plate. Cells were incubated in the dark for 2 hours, and then the absorbance was detected at a wavelength of 450 nm. At least 3 replicate wells were required for the same sample.

### Colony Formation Assay

FRH0201 and HuCCT1 cells were seeded in 6-well plates (500 cells/well), and the culture medium was changed every 4 days, washed with PBS after 2 weeks, fixed with methanol and stained with 0.5% crystal violet; cell colony formation was observed using an inverted microscope.

### Apoptosis Assay

Flow cytometry was used to detect the apoptosis of cells after drug addition. Flow cytometry: centrifuge cells to be tested for 5 min (1000 r/min) and discard the culture medium; rinse cells twice with 1 mL of precooled PBS, centrifuge for 5 min (1000 r/min) and discard the PBS, and collect 1 - 5 × 10^5^ cells; after re-suspension with 500 μL of precooled Binding Buffer, add 5 μL of AnnexinV-EGFP, and mix thoroughly, then add 5 μL of Propidium Iodide, mix well; cells react at room temperature for 10 min in the dark, and then were detect with Attune NxT flow cytometer (Thermofisher, USA).

### Western Blot Assay

The levels of BAX, BID, BIM, Caspase-3, Caspase-9, LC3-I, LC3-II and p62 were measured by Western blot. Protein extraction: adherent cells were collected by digestion and centrifugation (800 r/min × 4 min); these cells were resuspended by pre-cooled PBS, slowly blown and centrifuged (800 r/min × 4 min) to discard PBS, and washed three times repeatedly; cells were resuspended by protein lysis solution (IP lysis solution: PMSF = 100:1) and lysed in an ice box for 30min, followed by centrifugation (12000r/min×5min) at 4°C to obtain the supernatant; the supernatant concentration was measured by BCA kit (Shanghai Jining Biotechnology Co., Ltd., China). Protein separation: the protein extract was mixed with 5 × SDS-PAGE loading buffer in a ratio of 1:4, and the resulting solution was boiled at 100°C for 5 min; the protein was separated by 10% separation gel, and the voltage was changed from 80 volts (V) to 120 volts (V) after the protein marker was completely separated; the protein in the separation gel was transferred to the activated nitrocellulose membrane by semi-dry semi-wet method (4°C, 250 mA, 100 min); after the completion of transfer membrane, the membrane was blocked with skimmed milk for 1 hour, and washed with TBST every 5 minutes for 3 times; the membrane was incubated with primary antibody on a shaker (4°C, overnight); the membrane was washed with TBST every 5 minutes for 3 times and then incubated with secondary antibody (25°C, 2 hours); after washing with TBST every 5 minutes for 3 times, the membrane was developed using xiECL chromogenic solution in a darkroom, and then developed and exposed.

### Mouse Tumorigenesis Assay

FRH0201 cells were inoculated subcutaneously in nude mice, and 20 nude mice with similar tumor size were selected and randomly divided into five groups, 5 mice in each group. The drug was administered every other day *via* the tail vein. At 24 hours after dosing, PDT treatment was administered for 20 min, with a light source 2 cm from the tumor, a light intensity of 120 J cm^−2^, and a power of 0.2 W. Tumor size was monitored at fixed period, and weight of nude mice was recorded. The tumor growth curve and mouse weight curve were plotted according to the actual situation (about 3 weeks). Animal care and method procedure were authorized by the Animal Ethics Committee of Cangzhou Central Hospital.

### Immunohistochemistry

The mice were sacrificed by neck strangulation, and the removed tumor tissues were used for paraffin embedding and sectioning (thickness of 4 µm). Tissue sections were heated with Tris-EDTA buffer (pH = 9) at 120°C for 3 min in a pressure cooker and then treated with ethanol-dissolved 0.3% hydrogen peroxide for 20 min to block endogenous enzyme activity. Tissue sections were washed with PBS and blocked with 10% skimmed milk containing primary antibody for 1 hour at room temperature; after washing three times with PBS, tissue sections were incubated with secondary antibody for 45 min; after washing three times with PBS, tissue sections were incubated with avidin-biotin peroxidase complex for 45 min; these sections were reacted with peroxidase using a diaminobenzidine substrate kit; later, sections were stained with hematoxylin for 2 min, dehydrated with ethanol, dehydrated with xylene, and mounted. Finally, these sections was observed by a light microscope (Nikon, Japan), and calculated for their Ki67 positive cells.

### TUNEL Staining

Mouse tissue sections were taken for Tunel staining. Tissue sections were deparaffinized and hydrated with gradients of xylene and ethanol (100, 90, 80 and 70% ethanol); 20 μg/mL proteinase K was added dropwise to the sections, permitted to react at 37°C for 30 min and washed three times with PBS; 50 μL of TUNEL staining solution was added to each section and incubated at 37°C for 60 min under light-proof conditions; the sections were washed with PBS, added with termination solution and incubated for 10 min at room temperature; after washing three times with PBS, the sections was added dropwise with DAB color development solution and incubated for 10 min at room temperature; after washing three times with PBS, the nuclei were stained with hematoxylin staining solution, and the sections were sealed after washing three times with PBS for observation. Ten randomly selected fields per section were used to calculate the number of TUNEL-staining positive cells.

### Data Processing

The experiments involved in this study were performed in triplicate independently. The experimental data were statistically analyzed and processed by SPSS21.0 software, and GraphPad Prism 7 (GraphPad Software, San Diego, USA) was used to picture this data. Enumeration data were analyzed by χ2 test; measurement data were analyzed by ANOVA analysis of variance, and the differences between multiple groups were tested by Bonferroni test. P < 0.05 signified that the differences reached statistical significance.

## Results

### Structural Characterization of OH-MSNs

**Figure 1:**
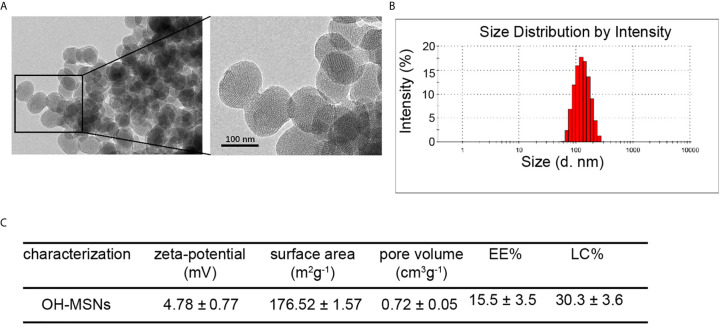
OH-MSNs characterization. **(A)** The structure of OH-MSNs was assessed by transmission electron microscope (TEM). **(B)** The size distribution of OH-MSNs was analyzed by Dynamic Light Scattering (DLS). **(C)** The characterizations, including zeta-potential, surface area, pore volume, EE% and LC%, of OH-MSNs were shown.

The OH-MSNs were successfully synthesized and the characterization results of OH-MSNs by transmission electron microscopy (TEM) (**Figure 1A**). The dynamic light scattering (DLS) analysis displayed that OH-MSNs nanoparticles had uniform spherical shapes with diameter about 120nM (**Figure 1B**). Moreover, the zeta-potential of the nanoparticles was 4.78 ± 0.77 mV (**Figure 1C**). The surface area of the nanoparticles was 176.52 ± 1.57 m^2^g^-1^ (**Figure 1C**). The pore volume was 0.72 ± 0.05 cm^3^g^-1^ (**Figure 1C**). The EE% and LC% was 15.5 ± 3.5% and 30.3 ± 3.6%, respectively (**Figure 1C**).

### Drug Release Profile

**Figure 2:**
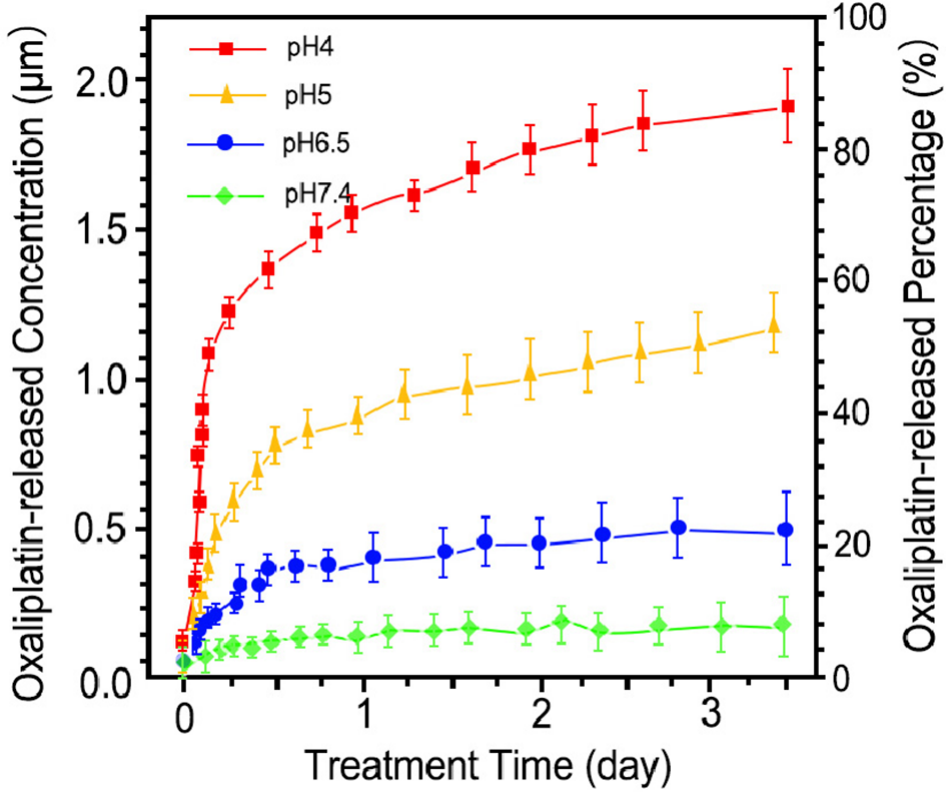
Drug release pattern of Oxaliplatin/HCE6-MSNs. The drug release analysis was performed at pH 4, pH 5, pH 6.5, and pH 7.4.

The release pattern of HCE6 from OH-MSNs at different pH is shown in **Figure 2**. Over time, HCE6 showed different degrees of sustained release at different pH. The efficiency of Oxaliplatin release by OH-MSNs increased with decreasing solution pH, and the release of Oxaliplatin by OH-MSNs was promoted under acidic conditions.

### The Cellular Uptake of OH-MSNs

**Figure 3:**
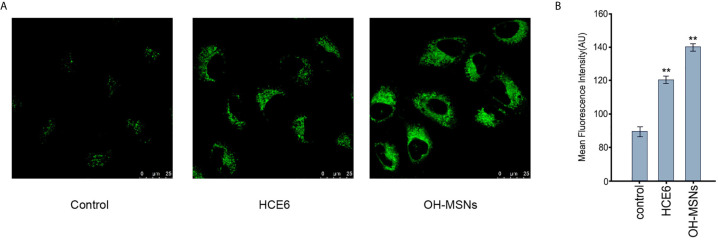
The cellular uptake of OH-MSNs. **(A, B)** The cellular uptake of OH-MSNs was analyzed by confocal fluorescence microscope in FRH0201 cells. **P < 0.01.

Next, the cellular uptake of OH-MSNs was analyzed by confocal fluorescence microscope in FRH0201 cells. Our data showed that OH-MSNs was able to be effectively uptake by FRH0201 cells (**Figures 3A, B**).

### Effects of OH-MSNs on the Proliferation and Viability of Cholangiocarcinoma Cells

**Figure 4:**
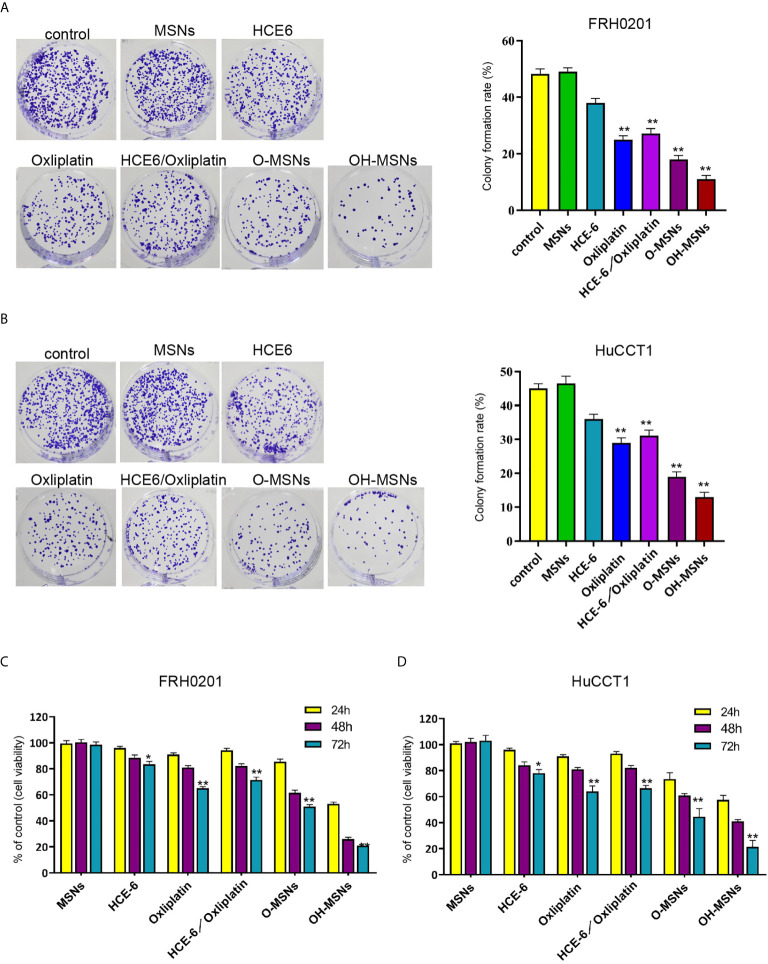
Cell viability and proliferation ability of cholangiocarcinoma cells. **(A–D)** The FRH0201 and HuCCT1 cells were treated as the indicated labeling. **(A, B)** The cell proliferation was measured by colony formation assays. **(C, D)** The cell viability was analyzed by CCK-8 assays. Data are presented as mean ± SD. Statistic significant differences were indicated: *P < 0.05, **P < 0.01.

The changes of FRH0201 and HuCCT1 cell activity and proliferative capacity after treatment for 24, 48 and 72 hours in different ways are shown in **Figure 4**. The results revealed that MSNs had no significant effects on the activity and proliferative capacity of FRH0201 and HuCCT1 cells (P>0.05), whereas both HCE6 and Oxaliplatin decreased the activity and proliferation of FRH0201 and HuCCT1 cells (P<0.05). Besides, the inhibitory effect of Oxaliplatin on FRH0201 and HuCCT1 cell activity and proliferation was significantly enhanced by MSNs loading, and the inhibitory effect of O-MSNs loaded with the photosensitizer HCE6 on FRH0201 and HuCCT1 cell activity and proliferation was far more effective in relative to O-MSNs (P>0.05).

### Effects of OH-MSNs on the Level of Apoptosis in Cholangiocarcinoma Cells

**Figure 5:**
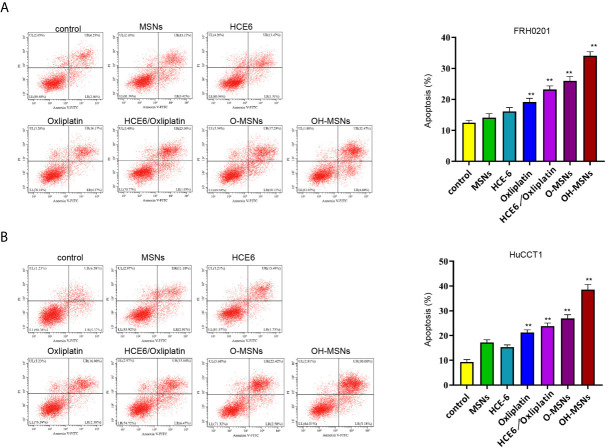
Apoptosis level of cholangiocarcinoma cells. **(A, B)** The FRH0201 and HuCCT1 cells were treated as the indicated labeling. The apoptosis was analyzed by flow cytometry. Data are presented as mean ± SD. Statistic significant differences were indicated: **P < 0.01.

The apoptosis level of FRH0201 and HuCCT1 cells treated in different ways is displayed in **Figure 5**. It was proven by flow cytometry that the apoptosis level of FRH0201 and HuCCT1 cells rose sharply after treatment with OH-MSNs (P < 0.05). Additionally, MSNs loading remarkably enhanced the killing effect of Oxaliplatin on FRH0201 and HuCCT1 cells (P < 0.05), while loading HCE6 on this basis could further enhance the killing effect of the drug on FRH0201 and HuCCT1 cells (P < 0.05).

### Effects of OH-MSNs on the Expression Levels of Autophagy and Apoptosis-Related Proteins in Cholangiocarcinoma Cells

**Figure 6:**
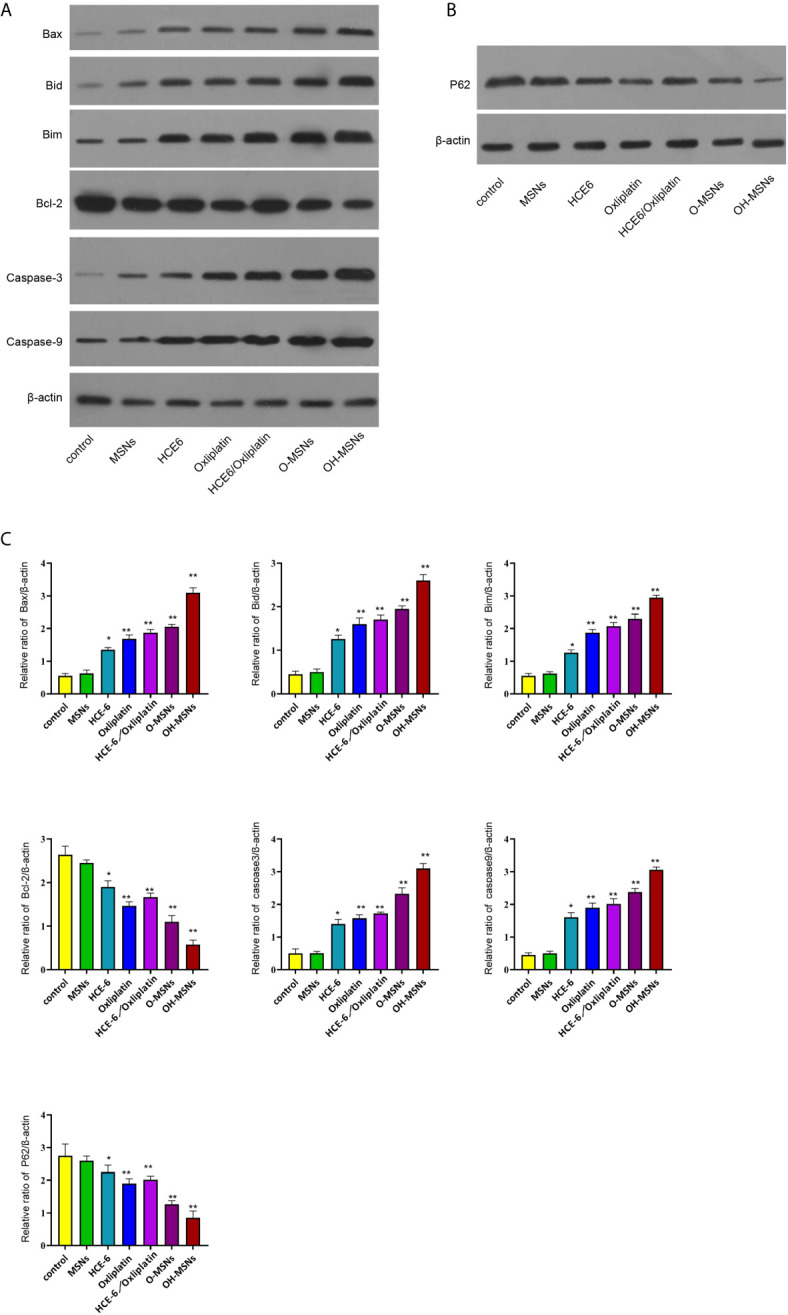
Expression levels of autophagy and apoptosis-related proteins in bile duct cancer cells. **(A–C)** The FRH0201 cells were treated as the indicated labeling. **(A)** The expression of Bax, Bid, Bim, Bcl-2, caspase3, caspase9 was measured by Western blot analysis. **(B)** The expression of P62 was detected by Western blot analysis. **(C)** The quantification of the Western blot analysis in A and B was analyzed by ImageJ software. Data are presented as mean ± SD. Statistic significant differences were indicated: *P < 0.05, **P < 0.01.

The expression levels of autophagy and apoptosis-related factors in FRH020 cells treated with different drugs are shown in **Figure 6**. It was found by Western blot that O-MSNs treatment was associated with notably increased expression level of pro-apoptotic factors such as Bax, Bid and Bim and apoptotic factors such as Caspase-3 and Caspase-9 in FRH0201 cells as well as suppressed expression level of anti-apoptotic factor BCL2 as compared to HCE-6 or Oxaliplatin (P<0.05). Furthermore, OH-MSNs further promoted the expression levels of pro-apoptotic factors, autophagy-promoting factors and apoptotic factors, and suppressed the expression levels of anti-apoptotic factors and autophagy-inhibiting factors compared with O-MSNs (P<0.05).

### Effects of OH-MSNs on Tumor Growth in Mice

**Figure 7:**
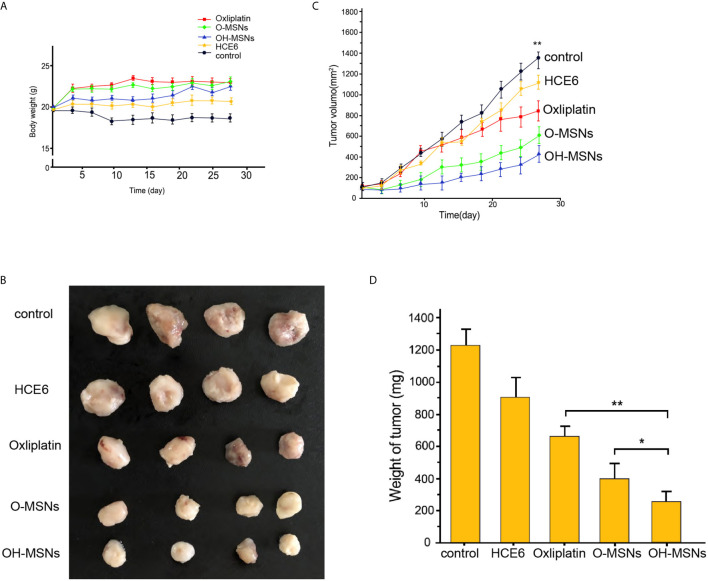
Tumor growth in mice. **(A–D)** The nude mice (n=4) were injected with FRH0201 cells and were treated as the indicated labeling. **(A)** The body weight was remarketed. **(B)** The tumor size was shown. **(C)** The tumor volume was shown. **(D)** The tumor weight was shown. Data are presented as mean ± SD. Statistic significant differences were indicated: *P < 0.05, **P < 0.01.

**Figure 8:**
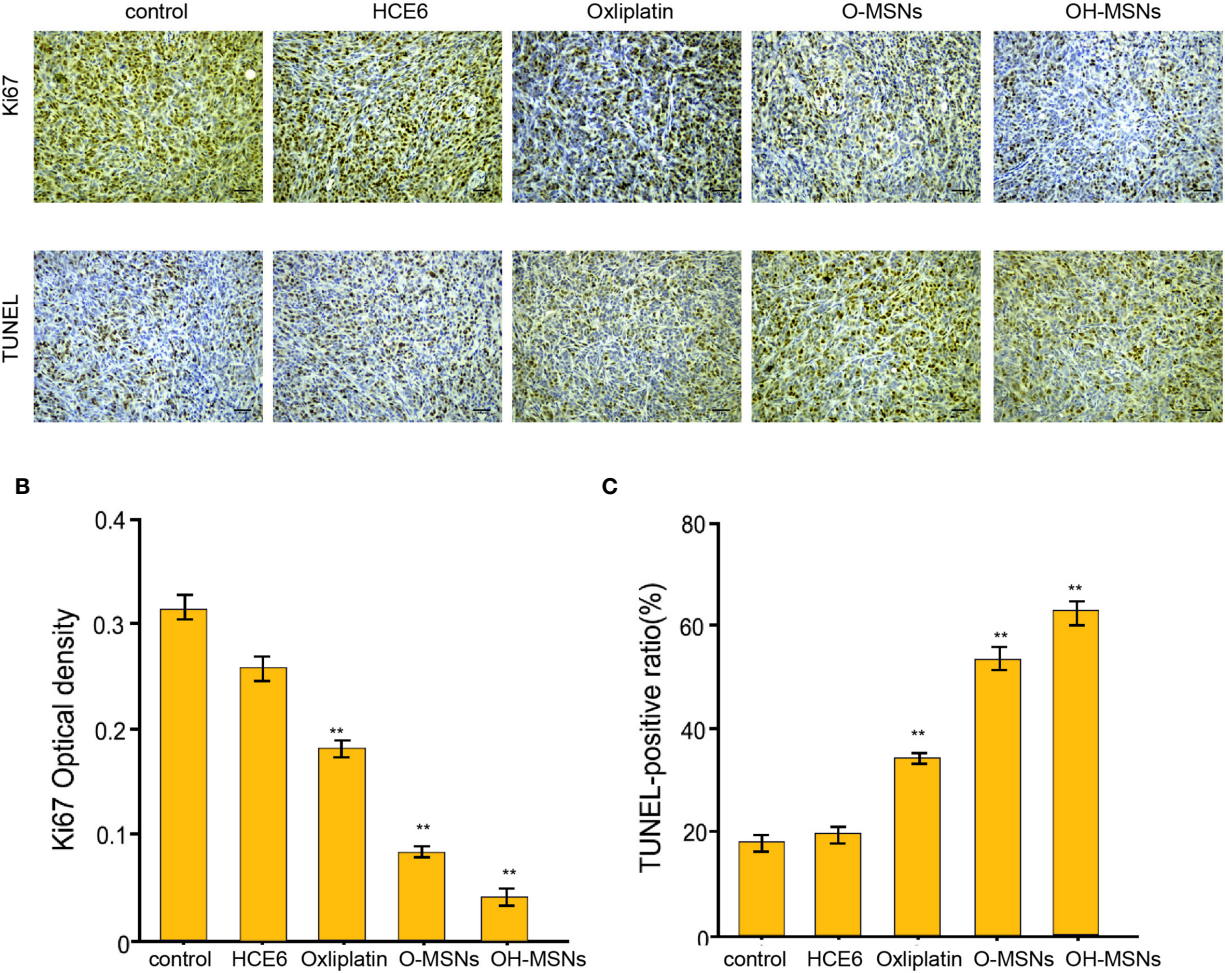
Ki67 and Tunel staining of mouse tumor sections. **(A–C)** The nude mice (n=4) were injected with FRH0201 cells and were treated as the indicated labeling. **(A, B)** The levels of ki-67 were measured by IHC. **(A, C)** The apoptosis was detected by TUNEL analysis. Data are presented as mean ± SD. Statistic significant differences were indicated: **P < 0.01.

The effects of different modes of administration on tumor growth in mice are shown in **Figures 7** and **8**. It was noted that Oxaliplatin showed a significant inhibitory effect on tumor growth in mice (**Figure 7**, P<0.05), while the volume and mass of tumors in mice were distinctly reduced in comparison with those treated by O-MSNs (**Figure 7**, P<0.05). OH-MSNs were much more effective in inhibiting tumor growth in mice compared with O-MSNs (**Figure 7**, P<0.05). In addition, Ki67 and Tunel staining results revealed that Ki67 levels in tumor cells of mice dropped significantly and the number of cells in the early stage of apoptosis increased markedly after treatment with O-MSNs (**Figure 8**, P<0.05), but the decline in Ki67 levels in mice cells was even more dramatic and the number of cells in the early stage of apoptosis was much more prominent after treatment with OH-MSNs (**Figure 8**, P<0.05).

## Discussion

Bile duct cancer is a common malignant tumor of the digestive system with a high incidence in China as well as in certain Southeast Asian countries (18). As medical technology is constantly improving, more and more therapeutic strategies have been utilized in the treatment of cholangiocarcinoma. Nevertheless, the overall prognostic survival rate of patients with bile duct cancer is still unsatisfactory (19). Currently, the treatment of bile duct cancer mainly adopts radiotherapy, chemotherapy and surgical intervention (1, 20). The chemotherapeutic drugs selected in clinical practice usually have strong toxic side effects, and patients usually suffer from gastrointestinal adverse effects such as nausea and vomiting after receiving a period of treatment. In addition, Oxaliplatin also has certain neurotoxicity, and even triggers symptoms such as numbness in the end of hands and feet in patients (21). As research has progressed, studies have shown that drug carrier-mediated delivery alters drug distribution in human, affects local concentrations by controlling the rate of drug release, or targets drug transport to patient foci (22). MSN has been extensively adopted as a promising drug delivery material over the past decades. The abundant surface area and specific straight channels of MSN facilitate the loading of various molecules including drugs, RNAs and DNAs (23). Moreover, MSN also exhibit a superior ability in controlling drug release due to the simple attachment of drugs in the mesopores, and the adjustable size and shape of the mesopores. Lv et al. (24) revealed in their study that MSNs loading was effective in reducing the toxic effects of cisplatin on the heart, liver, and kidneys of patients as well as the adverse effects during chemotherapy in cancer patients. In this study, OH-MSNs drug delivery system was synthesized using MSNs loaded with Oxaliplatin and photosensitizer HCE6, and a novel dosing strategy was proposed. Meanwhile, the effect of OH-MSNs on the cell activity, proliferation ability, apoptosis level, clone formation ability and intracellular apoptosis and autophagy-related factor expression levels of cholangiocarcinoma FRH0201 was investigated intensively in this study, and the inhibitory effect of OH-MSNs on cholangiocarcinoma was further analyzed by tumorigenesis experiments in mice. Moreover, even though HCE6 alone neither significantly affect cell proliferation nor synergize with oxaliplatin *in vitro* and *in vivo*, we found that the HCE6 and oxaliplatin loaded in MSN exhibited notably stronger cytotoxicity than naked HCE6 and oxaliplatin, these findings suggested the advantage of this MSN drug delivery system.

In this study, it was found that there was a link between the drug sustained-release rate and the pH in the drug microenvironment after loading by MSNs, and the drug release rate of O-MSNs rose dramatically with the decrease in environmental pH. Besides, the drug release rate of synthesized OH-MSNs remained higher in the environment with lower pH but lower in neutral conditions. The possible reasons for these pH-related drug release may be caused by the altered electrostatic repulsion due to repulsion between the drug and the OH-MSNs (25). Meanwhile, we investigated the drug release rate of O-MSNs in PBS buffer, and we will validate the results in other buffers, such as borate and acetate in future studies. The pH of the normal tissue microenvironment is usually neutral or weakly alkaline, whereas the internal environment of tumor tissue is generally acidic. Excessive aerobic glycolysis is one of the main characteristics of cancer cells (26). Cancer cells proliferate at a faster rate and have a greater demand for energy, which makes it difficult to obtain large amounts of energy with aerobic respiration in normal cells, while the glycolytic pathway allows cancer cells to be free from limitation of oxygen content. Therefore, glycolysis is a primary way to obtain energy by cancer cells (27). Lactate is one of the metabolites of the cellular glycolysis (28). As cancer cells metabolize a large amount of lactic acid, the pH of their surrounding microenvironment is low relative to the normal tissue environment (29). In this study, we found that MSNs loaded with Oxaliplatin increased considerably the killing effect of Oxaliplatin on tumor cells compared with Oxaliplatin alone, suggesting that the sustained release property of OH-MSNs may upgrade the drug level in cancer tissue sites. In addition, Li et al. (30) demonstrated that the rate of adriamycin release from the adriamycin-MSNs system was also significantly correlated with pH and that the system exhibited a higher rate of adriamycin release when it was in an acidic environment and was able to exert a better killing effect on tumor cells.

Based on O-MSNs, the present study further investigated the cancer inhibitory effect of OH-MSNs. The results showed that compared with O-MSNs, the intervention of OH-MSNs combined with light promoted the expression of apoptotic factors in cholangiocarcinoma cells and the apoptosis of cancer cells distinctly. PDT is an emerging mode for cancer treatment. It refers to the ability of photosensitizers to promote oxidative cellular damage by elevating intracellular free radical levels under light conditions, inhibiting tumor growth and improving patients’ symptoms (31). Photosensitizers are indispensable for PDT. Under light conditions, photosensitizer molecules in the excited state interact with substances such as oxygen and water molecules inside tissues to form free radicals, which contribute to cancer cell damage and apoptosis by elevating intracellular oxidative stress levels (32). However, electrons in the excited state in photosensitizers are usually unstable and prone to be trapped by electron holes in molecules inside or outside and reattributed to the ground state, thus disfavoring the formation of free radicals (33). It has been shown that materials such as MSNs can retard the electron-hole complexation rate of photosensitizers, and the overall charge separation effect of the system formed after the loading of photosensitizers by MSNs is significantly increased, thus favoring the formation of free radicals (34). Additionally, Zhang et al. revealed (35) that MSNs loading under constant light source conditions significantly enhanced the capability of the photosensitizer CE6 to form active oxygen, and that the mode of administration of CE6-MSNs versus CE6 was correlated with notably elevated level of oxidative stress in tumor cells, and even increased killing effect of the drug on tumor cells. Moreover, basing on the enhanced permeability and retention (EPR) theory (36) that the circulating nanoparticles are easily accumulated in tumor areas, the OH-MSNs are capable of exhibiting higher cytotoxicity comparing with single HEC6 or Oxaliplatin.

Autophagy, one of the important cellular activities, usually emerges when the cellular energy supply is insufficient, or the cell is in a stressful state (37). Studies have shown that appropriate levels of autophagy increase cellular activity and facilitate cellular protection from toxins, oxidative stress, and other damage, which has positive implications for cell survival, while excessive autophagy may also lead to apoptosis (17). Elevated levels of intracellular oxidative stress are one of the major causes of cellular autophagy. Yang et al. (38) found that the level of oxidative stress was remarkably elevated in gastric cancer cells of mouse after treatment with ZnO nanoparticles, along with a significant increase in intracellular autophagic vesicles, leading to an elevated level of cellular autophagy and even inducing apoptosis in cancer cells. Besides, it has also been revealed that drugs such as Oxaliplatin induce apoptosis in cancer cells by increasing the level of cellular autophagy (39). In this study, we found that the dramatic changes in expression levels of autophagy-related factors (LC3-I), and notably increased levels of pro-apoptotic factors (Bim) were observed in cholangiocarcinoma FRH0201 after treatment with OH-MSNs. Furthermore, the effects of OH-MSNs on the autophagy level of cholangiocarcinoma FRH0201 were more salient compared with Oxaliplatin. We drew a conclusion by these results that OH-MSNs increased the level of cellular autophagy to induce apoptosis in cholangiocarcinoma cells.

In summary, this study confirmed that OH-MSNs show a stronger inhibitory effect on cholangiocarcinoma compared with single Oxaliplatin. Moreover, it promotes apoptosis of cholangiocarcinoma cells and inhibits the growth of cholangioma by increasing the level of intracellular autophagy.

## Data Availability Statement

The original contributions presented in the study are included in the article/supplementary material. Further inquiries can be directed to the corresponding author.

## Ethics Statement

The animal study was reviewed and approved by Cangzhou Central Hospital.

## Author Contributions

PZ performed the majority of experiments and analyzed the data. FF performed the molecular investigations. ML designed and coordinated the research. KL wrote the paper. All authors contributed to the article and approved the submitted version.

## Conflict of Interest

The authors declare that the research was conducted in the absence of any commercial or financial relationships that could be construed as a potential conflict of interest.
